# Clinical utilization of serum- or plasma-based miRNAs as early detection biomarkers for pancreatic cancer: A meta-analysis up to now

**DOI:** 10.1097/MD.0000000000012132

**Published:** 2018-08-21

**Authors:** Lunshou Wei, Kunhou Yao, Shibao Gan, Zhimin Suo

**Affiliations:** a Department of Gastroenterology; b Department of General Surgery, Huaihe Hospital of Henan University, Henan Province, China.

**Keywords:** diagnosis, meta-analysis, micro-RNA, pancreatic cancer

## Abstract

**Background:**

Pancreatic cancer (PC) is a lethal disease, however current screening methods unable to achieve early diagnosis. Blood-based microRNAs (miRNAs) are promising molecular biomarkers for detecting PC. This meta-analysis summaries studies identifying serum- or plasma-based miRNAs dysregulated in PC patients compared to non-PC cases to evaluate their diagnostic accuracy for characterizing PC.

**Methods:**

A systematically reviews and meta-analysis of published studies was conducted to compare the serum or plasma miRNAs expressions between PC patients and non-PC cases. Summary estimates for sensitivity, specificity, along with other measures of accuracy of miRNAs in the diagnosis of PC were pooled using the random-effects model. *I*^*2*^ and *Q* tests were used to assess the heterogeneity of included studies. The Spearman test was used to analyze the threshold effect.

**Results:**

Twenty-seven eligible studies were identified after electronic search and literature selection. For single miRNA dysregulation, 32 miRNAs were found to be upregulated in PC patients, and 5 miRNAs were downregulated. Four studies identified a 2-miRNA panel, and 10 studies identified a panel consisting of 3 or more miRNAs which were used to detect PC patients. Additionally, 8 studies combined miRNA panels and carbohydrate antigen 19–9 (CA 19–9) to diagnose PC. The pooled sensitivities for these 4 groups were 0.77 to 0.85, and specificities were 0.70 to 0.87. The highest area under the curve (AUC), 0.9308, was identified using 2 miRNA panels with sensitivity and specificity of 0.79 (0.74–0.83) and 0.85 (0.81–0.89), respectively. There was great heterogeneity of these 4 miRNA groups. Results of Spearman test revealed that there existed a threshold effect on single miRNA group (*r*=−0.437, *P*=.001), and none of the other groups (*P* all>.05).

**Conclusions:**

Serum- or plasma-based miRNAs are capable of distinguishing PC from non-PC with relatively high sensitivity and specificity. In future, miRNAs may be used as promising diagnostic biomarkers for detection of PC.

## 1 Introduction

Pancreatic cancer (PC) remains one of the most recalcitrant cancers, with a 5-year survival rate lower than 5%.^[1,2]^ Surgical resection constitutes the most effective strategy,^[3]^ due to its high resistance to chemotherapy and radiotherapy. Unfortunately, potentially resectable localized tumors are less than 25%.^[4]^ Based on the data, surgical resection of pancreatic cancers at early stage can lead to 2 more years survival,^[5]^ making it urgent to develop screening tool with simultaneously high sensitivity and specificity.

Conventional diagnostic methods including radiological imaging and serum markers.^[2]^ However, common imaging modalities (computed tomography [CT], magnetic resonance imaging [MRI], endoscopic ultrasound [EUS]) often starts only after the local and systemic symptoms appear, leading most PC patients already at advanced stage at initial diagnosis. As for carbohydrate antigen 19–9 (CA 19–9) often fail to detect precancerous or early stage lesion because of its inadequate sensitivity and specificity but are routinely used to assess known disease prognosis.^[6,7]^ For the past few years, significant efforts have been dedicated to seeking the novel biomarkers to help early diagnosis of PC.^[3,8]^ Further understanding of the processes that govern the development of PC is essential as it lights on potential biomarkers of early detection.

MicroRNAs (miRNAs) are single-stranded small RNA molecules with 19 to 25 nucleotides, which regulate genetic expression at the post-transcriptional level by binding to 3’ or 5’-untranslated regions of the targeted mRNA or the open reading frames.^[9,10]^ Through this interaction, miRNAs can lead to mRNA degradation or suppression of protein translation.^[3]^ The deregulation of miRNAs can be the consequence of gene mutations or deficiency in the miRNA processing pathway, resulting in the developing of substantial number of diseases, including cancers.^[11]^ miRNAs can be classified as either oncogenic or tumor suppressor according to their functions in the carcinogenic process in oncology.^[12]^ Circulating miRNAs are released from diseased tissues into circulation as part of the extracellular crosstalk between cells and function as hormone-like signals.^[11]^ Because miRNAs are very stable molecules, they can provide a readout of the tissue's steady state and serial analyses which will imply changes in disease state.^[11]^

Therefore, a slew of studies considered blood-based miRNAs as potential biomarkers of PC that could contribute to early diagnosis, as well as prediction of lesion progression.^[13,14]^ Nevertheless, different miRNAs have been investigated in a large number of studies that affected their comparability with respect to the diagnostic accuracy of PC. The purpose of this meta-analysis is to evaluate published studies using plasma- or serum-based miRNAs as biomarkers for the diagnosis of PC and to validate their capacities.

## 2 Materials and methods

This present meta-analysis was conducted following the PRISMA statement. This study was reviewed and approved by the ethics committee of Henan University.

### 2.1 Search strategy

A comprehensive search was performed to identify all studies that assessed the diagnostic accuracy of plasma- or serum-based miRNAs for PC in PubMed, EMBASE, and Cochrane Library up to November 25, 2017. Keywords including “plasma” or “serum”, “microRNA” or “miRNA” or “miR”, “pancreatic neoplasms” or “pancreatic cancer” or “pancreatic tumor”. Both Medical Subject Headings (MeSH) and freestyle words were searched. The reference lists of relevant studies and reviews were also searched for retrieving potentially eligible studies.

### 2.2 Inclusion/exclusion criteria

To be included, studies had to satisfy the following criteria:

(1) related to the diagnostic value of miRNAs for PC;
(2) miRNAs’ expression levels were detected in serum or plasma;
(3) all patients were diagnosed as PC by using gold standard test;
(4) sufficient data were provided to calculate estimates of true positives (TP), false positives (FP), true negatives (TN), and false negatives (FN).

Exclusion criteria:

(1) unrelated to diagnostic value of miRNAs for PC;
(2) duplicated publications or incomplete data;
(3) letters, reviews, case reports, and editorials;
(4) studies not performed on humans.

### 2.3 Data extraction

Two reviewers independently screened the titles, abstracts and full texts of qualified studies. The following data were extracted from eligible studies: first author, publication year, country, specimen, sample size (both cases and controls), miRNA profiling, test methods, specimen sources, TP, FP, TN, FN, and any other additional information required for quality evaluation.

### 2.4 Statistical analysis

Data analyses were undertaken using Meta-Disc statistical software (version 1.4, Universidad Complutense, Madrid, Spain) for Windows. Owing to the presumed heterogeneity of studies, the random-effects model was utilized to estimate the pooled sensitivity, specificity, positive, and negative likelihood ratio (PLR and NLR) and diagnostic odds ratio (DOR) along with their corresponding 95% confidence intervals (CIs) by the following formulas: sensitivity = TP/(TP+FN); specificity = TN/(FP+TN); PLR = sensitivity/(1-specificity); NLR = (1-sensitivity)/specificity; DOR = (TP×TN)/(FP×FN). The summary receiver operating characteristic curve (SROC) was plotted and area under the curve (AUC) was calculated to quantitatively measure the diagnostic accuracy. The heterogeneity of included studies was investigated using *I*^*2*^ and *Q* tests. The Spearman test was used to analyze the threshold effect. All *P* values were 2-sided. The quality of the eligible studies was assessed by the quality assessment of diagnostic accuracy studies (QUADAS) criteria.

## 3 Results

### 3.1 Search results

**Figure 1:**
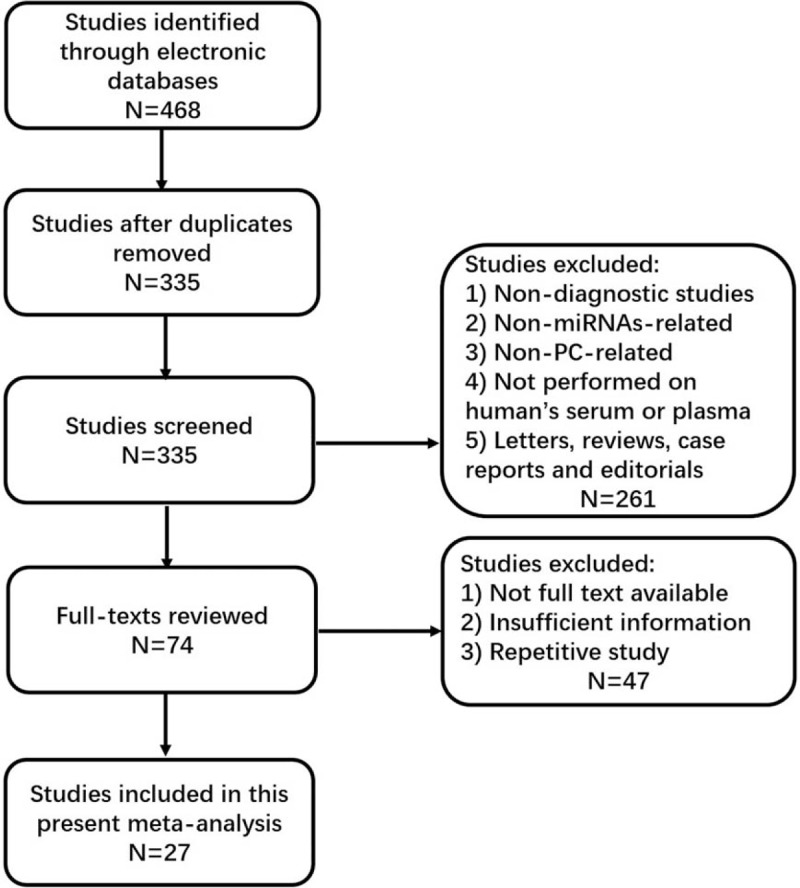
Flow chart of study selection.

With comprehensively literature selection process showed in Figure 1, 468 initial studies were obtained from 3 electronic databases. After importing all the studies into EndNote X7, 133 duplicated studies were excluded by automatically and manually duplicate checking procedure. The remaining 335 studies were screened, and after title along with abstract review, 261 studies were subsequently excluded owing to non-diagnostic studies, non-miRNAs related, non-PC-related, not performed on human's serum or plasma, and no original articles. Full texts were further screened for the remaining 74 potentially eligible articles. Afterward, 47 studies were excluded for the following reasons: no full texts available, insufficient information and repetitively studies. Thus, leaving 27 qualified original studies included for the final systematic review.^[15–41]^

### 3.2 Characteristics and quality assessment of eligible studies

As seen in Table 1, a total of 4909 subjects were included; 2413 PC patients, and 2496 non-PC patients (including patients with chronic pancreatitis, benign pancreatic tumors, other non-pancreatic cancers, pancreatic neuroendocrine tumors, diabetes, autoimmune pancreatitis), or healthy controls. All PC patients included in this meta-analysis were confirmed by histopathology, and patients with other diseases were confirmed by radiology imaging or clinical diagnosis with close follow-up. All of the eligible studies detected dysregulated miRNAs using quantitative real-time polymerase chain reaction (qRT-PCR) in serum (n=16) or plasma (n = 11). Among them, 10 studies evaluated single miRNA, and the other 17 for multiple miRNAs. Sixteen studies were conducted in Asian countries (China and Japan), and the rest 11 studies were in non-Asian countries (USA, Brazil, Czech, Denmark, Egypt, and Germany). Quality assessment results of included studies using QUADAS score were also shown in Table 1.

**Table 1:**
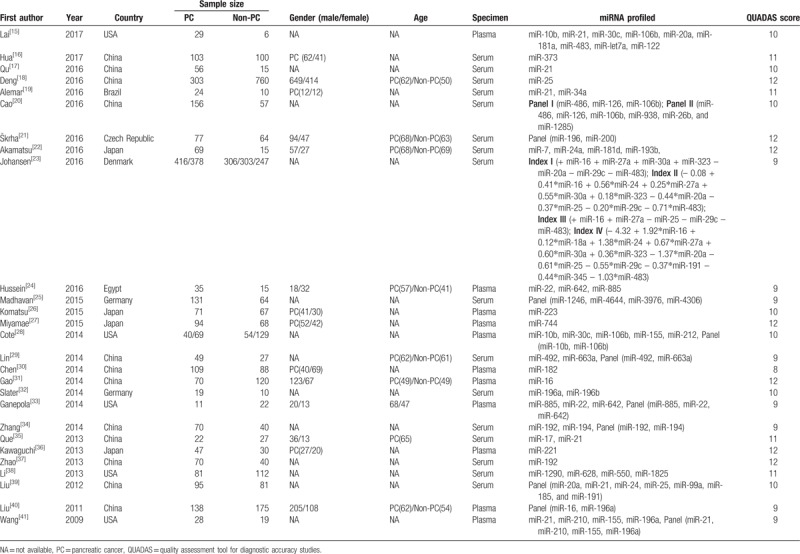
Characteristics and quality assessment of studies in the present meta-analysis.

### 3.3 Significantly dysregulated miRNAs in the plasma of serum of PC patients

When comparing single miRNA dysregulation, 32 miRNAs were found to be upregulated in PC patients when compared to non-PC patients or controls, and 5 miRNAs were downregulated. Eleven upregulated miRNAs (miR-10b, miR-21, miR-30c, miR-34a, miR-22, miR-642, miR-885, miR-155, miR-192, miR-196a) were identified by more than 1 study, among them, miR-21 was the most frequently identified dysregulated miRNA (seen in Table 2 ).

**Table 2:**
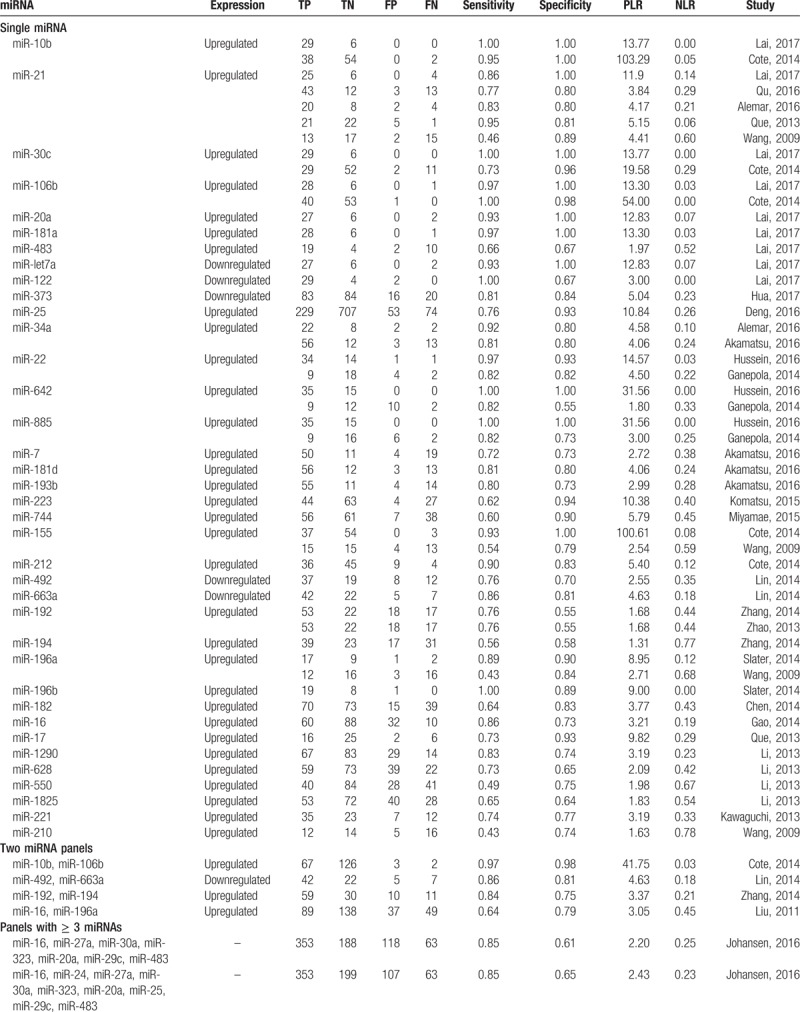
Significantly dysregulated miRNAs in PC patients’ plasma and serum as compared to non-PC patients.

**Table 2 (Continued):**
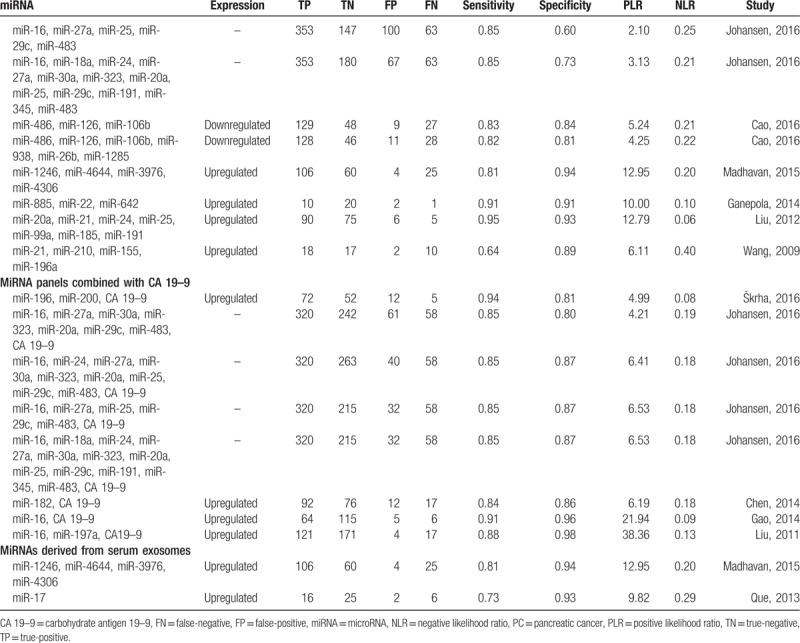
Significantly dysregulated miRNAs in PC patients’ plasma and serum as compared to non-PC patients.

Four studies identified a 2-miRNA panel, and 10 studies identified a panel consisting of 3 or more miRNAs which were used to detect PC patients. Additionally, 8 studies combined miRNA panels and CA 19–9 to diagnose PC. Moreover, among 16 studies using serum-derived miRNAs, 2 of them used exosomes-derived miRNAs (a panel of miR-1246, miR-4644, miR-3976, miR-4306, and miR-17) in particular (seen in Table 2 ).

### 3.4 Diagnostic capacity analysis

**Figure 2:**
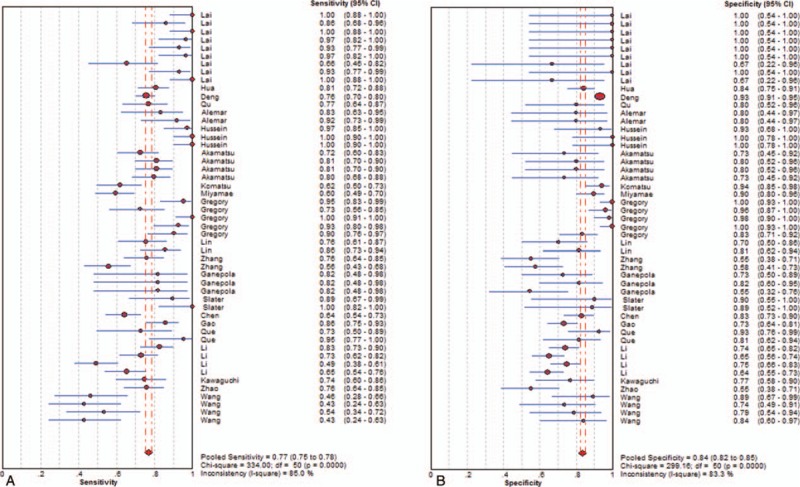
Sensitivity (A) and specificity (B) of diagnosis of PC with single miRNA. miRNA = microRNA, PC = pancreatic cancer.

**Figure 3:**
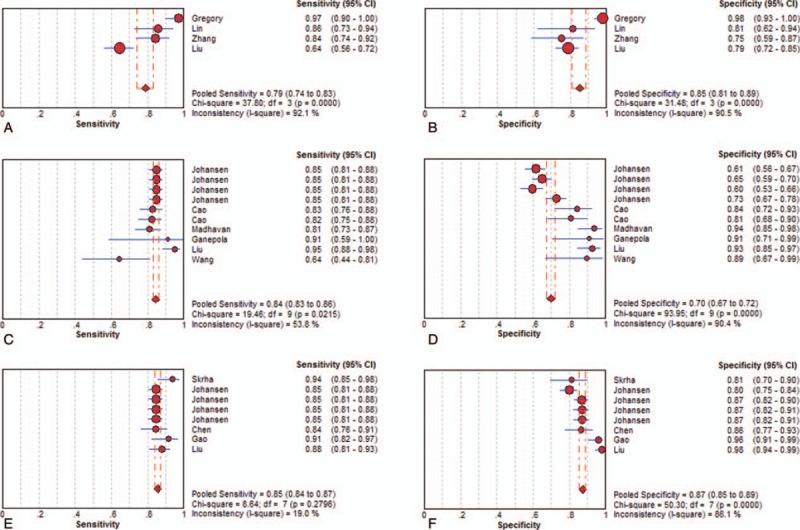
Sensitivity and specificity of diagnosis of PC with 2 miRNA panels (A and B), with panels with ≥ 3 miRNAs (C and D), and miRNA panels combined with CA 19–9 (E and F). CA 19–9 = carbohydrate antigen 19–9, miRNA = microRNA.

**Figure 4:**
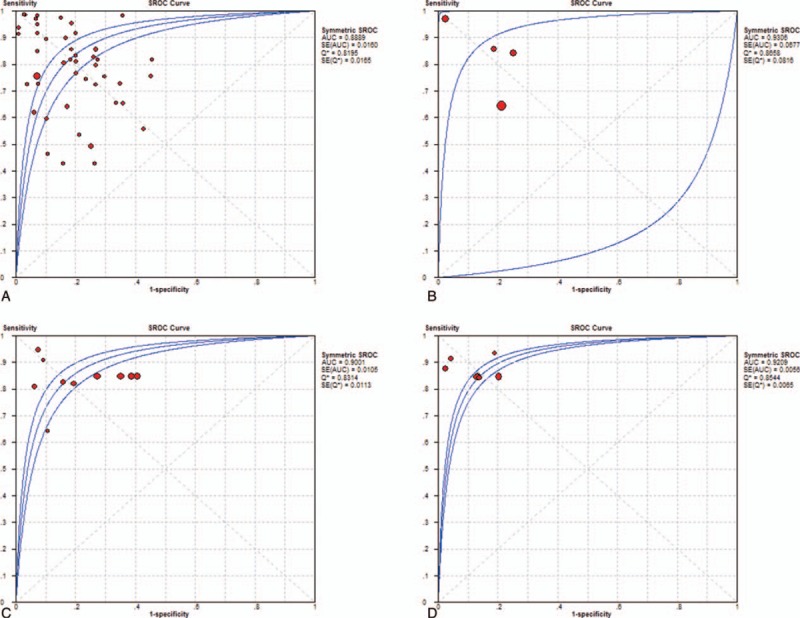
SROC curves of single miRNA (A), 2 miRNA panels (B), with panels with ≥ 3 miRNAs (C), and miRNA panels combined with CA 19–9 (D). CA 19–9 = carbohydrate antigen 19–9, miRNA = microRNA, SROC = summary receiver operating characteristic curve.

As seen in Figures 2–4, and Table 3, the sensitivity, specificity, PLR, NLR, DOR, and AUC for studies that used a single dysregulated miRNA in PC patients compared with non-PC cases were 0.77 (95% CI 0.75–0.78), 0.84 (95% CI 0.82–0.85), 4.08 (95% CI 3.21–5.19), 0.27 (95% CI 0.22–0.33), 18.62 (95% CI 12.54–27.64), and 0.8889. The ability to discriminate PC from non-PC cases of 2 miRNA panels was similar to single miRNA panel, with sensitivity, specificity, PLR, NLR, DOR, and AUC were 0.79 (0.74–0.83), 0.85 (0.81–0.89), 5.86 (2.46–13.94), 0.17 (0.07–0.46), 37.45 (6.78–206.86), and 0.9308, respectively. The sensitivity of ≥ 3 miRNA panels group was improved than single miRNA or 2 miRNA panels (0.84, 95% CI 0.83–0.86), while the specificity was decreased (0.70, 95 CI% 0.67–0.72), and the PLR, NLR, DOR, and AUC of it were 3.66 (95% CI 2.79–4.80), 0.22 (95% CI 0.19–0.26), 19.01 (12.08–29.93), and 0.9001. The combination of miRNA panels and CA 19–9 had the highest sensitivity (0.85, 95% CI 0.84–0.87), with good specificity, PLR, NLR, DOR, and AUR (0.87, 95% CI 0.85–0.89; 7.23, 95% CI 5.24–9.99; 0.17, 95% CI 0.15–0.19; 49.38, 95 CI% 31.09–78.42; and 0.9209). Despite only 2 studies used serum exosome-derived miRNA to distinguish PC from non-PC cases, its ability remained very high, with sensitivity, specificity, PLR, NLR, and DOR were 0.80 (95% CI 0.72–0.86), 0.93 (95% CI 0.86–0.98), 11.82 (95 CI% 4.99–33.56), 0.22 (95% CI 0.16–0.30) and 52.69 (95% CI 20.84–133.24), respectively.

**Table 3:**
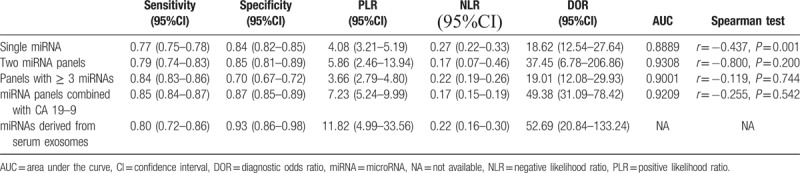
Summary diagnostic accuracy of serum- or plasma-based miRNAs for detecting PC.

### 3.5 Heterogeneity results

There was significant heterogeneity of all miRNA groups except for exosomes-derived miRNA panels. The Spearman test was used to analyze the threshold effect. And the results revealed that there existed threshold effect on single miRNA group (*r* = –0.437, *P* = .001), and none of the 3 groups (*P* all>.05) except for exosome-derived miRNA group (Table 3). Due to lots of factors influenced the miRNA extraction, normalization process, we were not able to conduct subgroup and regression analysis at present meta-analysis.

## 4 Discussion

PC is considered 1 of the most lethal cancers in the world, with the main cause of late detection.^[42]^ Very little progress has been made to improve the outcomes of advanced PC patients, which has motivated research in characterizing PC at early stage with novel and non-invasive means. Currently, endoscopic ultrasound-guided fine needle aspiration biopsy is widely used to obtain diagnostic material,^[43]^ however it suffered from compromised sensitivity, invasive procedure and unable to early diagnose. Various imaging technologies are also used to evaluate PC lesions, whereas it is difficult to distinguish benign or non-neoplastic mass from PC lesion to some extent, for instance, chronic pancreatitis can mimic PC on imaging.^[44]^ It's conceivable that blood samples can reflect the pathological processes with abundant miRNAs increased or decreased, leading to this “liquid biopsy” possible to detect PC situation.^[11]^ Moreover, collecting repeat serial blood samples has the potential to provide a molecular footprint of PC progress as well as monitor treatment responses in clinic with minimally invasive procedure.^[11]^

Abnormal alterations in miRNA expression are commonly associated with carcinogenic process of PC, including proliferation, invasion, apoptosis escape, metastasis, and epithelial-mesenchymal transition (EMT).^[45]^ In our present meta-analysis, a handful of dysregulated miRNAs were summarized in Table 2 . When focused on single miRNA dysregulation, 32 oncogenic miRNAs were found to be upregulated, and 5 antioncogenic miRNAs were downregulated in PC patients. Of which, mir-21 is the most widely studied miRNAs among all qualified original studies. High expression was described in 75% pancreatic ductal adenocarcinoma (PDAC), which is the most common type of PC.^[46]^ Over expressed miR-21 was correlated with downregulation of the tumor-suppressor genes TIMP3 and PDCD4, which resulting adverse course of PDAC.^[46]^ Additionally, miR-21 could cooperate with miR-23a and miR-21 as repressors of a network of antioncogenic genes (*BTG2*, *NEDDRL*, and *PDCD4*).^[47]^ The above studies all indicated that miR-21 played an integral role in tumor pathogenesis, which made it a potential biomarker in early detection.

Our meta-analysis revealed that a single dysregulated miRNA panel had moderate ability to discriminate PC from non-PC cases with sensitivity of 0.77 and specificity of 0.84. To further improve the diagnostic validity, many studies^[25,39,41]^ used miRNA panels to detect PC. However, our results showed only a little bit of progress had been made so far. Two miRNA panels were similar to single miRNA panel, with sensitivity and specificity of 0.79, 0.85, respectively. Sensitivity of ≥ 3 miRNA panels was improved than single miRNA or 2 miRNA panels (0.84), while the specificity was decreased (0.70). CA 19–9 is the most commonly used and most extensively validated serum biomarker for characterizing PC, but it is also elevated in a variety of other diseases, including other malignancies, for example, hepatocellular carcinoma and cholangiocarcinoma. According to studies, its sensitivity and specificity for detecting PC in symptomatic patients ranges from 79% to 81%, and 82% to 90%, respectively,^[48]^ due to lack of specificity. Thus, many studies^[31,40]^ committed to detect PC cases using combination of CA 19–9 and miRNAs. Our meta-analysis implied this combination could increase the diagnostic ability with sensitivity of 0.85 and specificity of 0.87.

During our data extraction process, 2 studies^[25,35]^ were found to use serum exosome- derived miRNAs to distinguish PC from non-PC cases with relatively high sensitivity and specificity (0.80 and 0.93). Exosomes are double-layer phospholipid membrane vesicles with small diameter of 30–100 nm.^[49]^ The biogenesis and trigger are strictly controlled by specific signaling molecules and activation of receptors with the function of mediating neighboring or long-distance cell-cell communications.^[50]^ Tumor-associated exosomes are detectable in serum, fostering the hope that circulating exosomal contents may be novel markers for PC screening and diagnosis. Exosome-derived miRNAs may have an advantage as biomarkers: living cell-secreted exosomal miRNAs can be found earlier in blood than necrosis-caused release of cell-free miRNAs, as the latter are usually increased at more advanced tumor stages.^[49]^ Although there are few studies focus on the exosomal miRNAs at present, along with the improvement and perfection of derived miRNAs procedure, further studies are likely to evaluate their diagnostic capacity of detection PC.

The main limitation of this meta-analysis is the great heterogeneity of all miRNA groups except for exosomes-derived miRNA panels. However, because of the complexity of included studies, the subgroup and meta-regression analysis were unable to conduct at the present stage. When assessing the threshold effect, we found out threshold effect on single miRNA group, this result further explained the different diagnostic subjects and standards might contribute to heterogeneity among studies.

Although our meta-analysis preliminary confirmed the utilization of blood-based cell-free miRNAs in detecting PC in clinic, there are still many challenges in identifying miRNA biomarkers for PC characterizing. Firstly, the standardization of miRNAs isolation from blood remains challenging. Secondly, the heterogeneity of PC or its immediate environment may result in heterogeneity of miRNAs in blood, leading compromised sensitivity and specificity of 1 or several miRNA panels. Additionally, from bench to bedside, the standard analysis platforms for miRNAs still remain before these biomarkers can be greatly used as a clinical tool.

## Author contributions

**Data curation:** Kunhou Yao, Shibao Gan.

**Methodology:** Lunshou Wei, Kunhou Yao.

**Project administration:** Shibao Gan, Zhimin Suo.

**Software:** Kunhou Yao.

**Visualization:** Shibao Gan, Zhimin Suo.

**Writing – original draft:** Lunshou Wei, Zhimin Suo.

**Writing – review & editing:** Lunshou Wei.
